# New Features on the Expression and Trafficking of mGluR1 Splice Variants Exposed by Two Novel Mutant Mouse Lines

**DOI:** 10.3389/fnmol.2018.00439

**Published:** 2018-12-03

**Authors:** Rika Naito, Hidetoshi Kassai, Yusuke Sakai, Sabine Schönherr, Masahiro Fukaya, Christoph Schwarzer, Hiroyuki Sakagami, Kazuki Nakao, Atsu Aiba, Francesco Ferraguti

**Affiliations:** ^1^Laboratory of Animal Resources, Center for Disease Biology and Integrative Medicine, Graduate School of Medicine, The University of Tokyo, Tokyo, Japan; ^2^Department of Pharmacology, Medical University of Innsbruck, Innsbruck, Austria; ^3^Division of Molecular Genetics, Kobe University Graduate School of Medicine, Kobe, Japan; ^4^Department of Anatomy, Kitasato University School of Medicine, Sagamihara, Japan; ^5^Laboratory for Animal Resources and Genetic Engineering, RIKEN Center for Developmental Biology, Kobe, Japan

**Keywords:** cerebellum, G protein-coupled receptor, CRISPR/Cas9, electron microscopy, glutamate, trafficking

## Abstract

Metabotropic glutamate receptors (mGluRs) couple to G-proteins to modulate slow synaptic transmission via intracellular second messengers. The first cloned mGluR, mGluR1, regulates motor coordination, synaptic plasticity and synapse elimination. mGluR1 undergoes alternative splicing giving rise to four translated variants that differ in their intracellular C-terminal domains. Our current knowledge about mGluR1 relates almost entirely to the long mGluR1α isoform, whereas little is known about the other shorter variants. To study the expression of mGluR1γ, we have generated by means of the CRISPR/Cas9 system a new knock-in (KI) mouse line in which the C-terminus of this variant carries two short tags. Using this mouse line, we could establish that mGluR1γ is either untranslated or in amounts that are undetectable in the mouse cerebellum, indicating that only mGluR1α and mGluR1β are present and active at cerebellar synapses. The trafficking and function of mGluR1 appear strongly influenced by adaptor proteins such as long Homers that bind to the C-terminus of mGluR1α. We generated a second transgenic (Tg) mouse line in which mGluR1α carries a point mutation in its Homer binding domain and studied whether disruption of this interaction influenced mGluR1 subcellular localization at cerebellar parallel fiber (PF)-Purkinje cell (PC) synapses by means of the freeze-fracture replica immunolabeling technique. These Tg animals did not show any overt behavioral phenotype, and despite the typical mGluR1 perisynaptic distribution was not significantly changed, we observed a higher probability of intrasynaptic diffusion suggesting that long Homers regulate the lateral mobility of mGluR1. We extended our ultrastructural analysis to other mouse lines in which only one mGluR1 variant was reintroduced in PC of mGluR1-knock out (KO) mice. This work revealed that mGluR1α preferentially accumulates closer to the edge of the postsynaptic density (PSD), whereas mGluR1β has a less pronounced perijunctional distribution and, in the absence of mGluR1α, its trafficking to the plasma membrane is impaired with an accumulation in intracellular organelles. In conclusion, our study sets several firm points on largely disputed matters, namely expression of mGluR1γ and role of the C-terminal domain of mGluR1 splice variants on their perisynaptic clustering.

## Introduction

The metabotropic glutamate receptor subtype 1 (mGluR1) is a G protein-coupled receptor (GPCR; Masu et al., 1991) that is involved in the regulation of neuronal excitability, synaptic plasticity and synapse elimination in the central nervous system (for review see Ferraguti et al., 2008). In the cerebellar cortex, mGluR1 is highly expressed in Purkinje cells (PCs) and regulate motor coordination, motor learning and long-term depression (Aiba et al., 1994; Conquet et al., 1994; Kano et al., 2008). Activation of mGluR1 stimulates phospholipase β4 (PLCβ4) via Gq protein (Miyata et al., 2001; Hartmann et al., 2004) and generates inositol 1,4,5-trisphosphate (IP_3_) and diacylglycerol (DAG; Aramori and Nakanishi, 1992). IP_3_ binds to IP_3_ receptor type 1 (IP_3_R1) on the endoplasmic reticulum (ER) membrane leading to Ca^2+^ release from the ER that in turn activates protein kinase Cγ (PKCγ; Finch and Augustine, 1998; Takechi et al., 1998). Activation of mGluR1 also leads to slow synaptic excitation that involves transient receptor potential channel 3 (TRPC3)-mediated cation influx (Hartmann et al., 2008).

Alternative splicing at the mGluR1 gene (*Grm1*) generates four translated variants, namely mGluR1α (a), mGluR1β (b), mGluR1γ (d) and mGluR1δ (E55), which share large part of the N-terminal sequence, but differ primarily in their intracellular C-terminal domain (Tanabe et al., 1992; Laurie et al., 1996; Zhu et al., 1999; Ferraguti et al., 2008). The mGluR1α isoform has the longest C-terminal domain and can physically interact with a variety of signaling, cytoskeletal and scaffolding proteins through motifs that are not present in the mGluR1β or mGluR1γ isoforms (Enz, 2012; Pin and Bettler, 2016; Suh et al., 2018).

PCs possess transcripts for both long, mGluR1α, and short, mGluR1β and mGluR1γ, variants (Tanabe et al., 1992; Berthele et al., 1998). Ultrastructural studies have shown that, at the subcellular level, both mGluR1α and mGluR1β preferentially accumulate perisynaptically (Baude et al., 1993; Nusser et al., 1994; Mateos et al., 2000; Techlovská et al., 2014; Mansouri et al., 2015). However, in the absence of the long mGluR1α isoform, mGluR1β displayed a more diffused distribution (Ohtani et al., 2014). Because of the lack of selective immunological tools, no information is available concerning the localization of the mGluR1γ isoform so far.

A huge influence on the mGluR1 trafficking and function was suggested for the binding of long Homer proteins to the C-terminus of the mGluR1α, that leads to the association with other signaling proteins such as the IP_3_R and Shank (Brakeman et al., 1997; Tu et al., 1998, 1999). We proposed that this interaction regulates the subsynaptic localization of mGluR1, but its disruption by the administration of the dominant-negative TAT-Homer1a protein did not result in any detectable change in mGluR1 distribution at parallel fiber (PF)-PC synapses (Mansouri et al., 2015). Although we were able to demonstrate that TAT-Homer1a could bind to mGluR1α, our study could not entirely rule out a contribution of long Homer proteins to the perijunctional accumulation of mGluR1.

In this study, we have generated a new knock-in (KI) mouse line using the CRISPR/Cas9 system in which the mGluR1γ carries two short tags allowing to study the expression of this variant in the cerebellum. In addition, we generated a second transgenic (Tg) mouse line carrying a point mutation in the Homer binding domain of mGluR1α to investigate the role of this interaction in the subcellular localization of mGluR1 at PF-PC synapses. We extended this analysis to other mouse lines selectively rescuing in mGluR1-knock out (KO) mice the expression of only one mGluR1 isoform in PCs to further explore the role played by the C-terminus of mGluR1 splice variants on their clustering in perisynaptic areas.

## Materials and Methods

All procedures involving animals were performed according to the methods approved by the animal care and use committee of The University of Tokyo Graduate School of Medicine and Kobe University Graduate School of Medicine. Mice were group-housed and kept in a climate-controlled room at 23 ± 1°C on a 12 h/12 h light/dark cycle and with free access to food and water within a specific pathogen-free (SPF) facility. Every effort was taken to minimize animal suffering and the number of animals used for this study.

### Generation of Genetically Modified Mice

The mGluR1-KO mice (originally kept on a mixed 129/Sv × C57BL/6 background) have been backcrossed more than eight times with C57BL/6. The C57BL/6N strain was used as wild type (WT) mice. We generated a new Tg mouse line (L7-mGluR1a-P/E) in which mGluR1α, carrying a point mutation, was reintroduced in mGluR1-KO under the control of the PC-specific L7 promoter (Oberdick et al., 1990) by means of previously described procedures (Ichise et al., 2000; Ohtani et al., 2014). The point mutation in the Homer binding domain of mGluR1α was introduced with the QuikChange Site-Directed Mutagenesis Kit (Agilent) replacing proline 1153 by a glutamate residue (P1153E). The mutant mGluR1α is referred to from now on as mGluR1α-P/E. For construction of the transgene, the rat mGluR1α-P/E complementary DNA (cDNA) was inserted into exon 4 of the L7 gene cassette. The transgene was microinjected into the pronuclei of fertilized mGluR1-KO embryos and four independent L7-mGluR1a-P/E Tg founder mice were obtained. For the generation of mGluR1γ-tagged KI mice, a single stranded oligodeoxynucleotide (ssODN) was synthesized to have the following sequence:

5′-GGGACAGCATGTGTGGCAGCGCCTCTCTGTGCACGTGAAGACCAATACCCATACGATGTTCCAGATTACGCTGGATCCGACTACAAGGATGACGATGACAAGTGAGACGGCCTGTAACCAAACAGCCGTAATTAAACCCCTCACTAA-3′

Cas9 protein (100 ng/μL), crRNA + tracrRNA (250 ng/μL) and ssODN (10 ng/μL) were mixed and microinjected into fertilized C57BL/6N embryos. Two-cell embryos were transferred into the oviducts of pseudo-pregnant female mice.

### RFLP Analysis

Genomic DNA was isolated from tail biopsies and amplified with the following primers: 5′-TCTGTGCAGGATCCATGTGT-3′ and 5′-GAACAAGGGCGTCTCTTCTG-3′; 10 μl of PCR product was digested with BamHI. The digested DNA was separated on an agarose gel (1.5%).

### Reverse Transcription PCR and Nested PCR for mGluR1 Splice Variants

For reverse transcription PCR, total RNA from mouse cerebellum was isolated with TRIzol (Invitrogen-Thermo Fisher Scientific). The obtained RNA was treated with recombinant DNase I (Takara Bio) and cDNA was synthesized with the RNA PCR Kit (AMV) Ver.3.0 (Takara Bio). The subsequent PCR was performed using the following primers:

forward    5′-GTGCCTTCACCACCTCTGAT-3′
reverse     5′-TGTAGTCGGATCCAGCGTAA-3′

For nested PCR, the following cDNAs were used as template for the PCRs: mouse cerebellar cDNA (Crepaldi et al., 2007), mouse whole brain Marathon-Ready cDNA (Clontech), rat cerebellar cDNA (Corti et al., 1998) and human cerebral cortex Marathon-Ready cDNA (Clontech).

PCR primers for mouse, rat and human were designed based on the following deposited sequences: NM_016976.3, M61099.1, L76627.1, respectively.

For the first PCR the following primers were used:

mouse—

forward    5′-CTGGGCTGCATGTTCACTCCCAAGAT-3′
reverse     5′-ATTGGTCTTCACGTGCACAGAGAGGC-3′

rat—

forward    5′-GGCCCTGGGGTGCATGTTTACTC-3′
reverse     5′-TGGTTACAGGCCGTCTCGTTGGTC-3′

human—

forward    5′-GTGCATGTTCACTCCCAAGATGTA C-3′
reverse     5′-TCTTCACGTGCACAGAGAGGCGGTG-3′

The amplification was carried out for 36 cycles of 30 s at 94°C denaturation and an annealing step at 65°C for rat and 62°C for mouse and human for 30 s followed by a polymerization step for 45 s at 72°C. The PCR products were resolved on a 2% agarose gel stained with Nancy-520 (Sigma) and analyzed using an E-Box imaging System (Vilber Lourmat CX5). From the obtained PCR products, bands between 200 bp and 400 bp were excised, gel purified and then diluted 1:500 to be used as template for nested PCRs using the following forward primers:

mouse     5′-GCCTGTCTTCCACTTGGCGTTCCCTG-3′
rat           5′-GCCTGTCTTCCACTTGGCATTCCCTG-3′
human     5′-ACCTGTCCTCCACTTGGCATTCCCTG-3′

The PCR amplification reaction was performed for 30 cycles of 30 s at 94°C, 30 s at 66°C and 45 s at 72°C. The PCR products were separated on a 2.5% agarose gel stained with Nancy-520 (Sigma-Aldrich).

### Rotarod

Motor coordination was assessed using the accelerating rotarod (Muromachi Kikai) based on previously described methods (Ohtani et al., 2014). Mice were placed on the rotating rod (3 cm in diameter), which was gradually accelerated from 4 to a maximum of 40 rpm over 5 min. The latency to fall was used as a measure of motor coordination and dexterousness.

### Immunoblot Analysis and Co-immunoprecipitation

Mouse cerebella were isolated and homogenized in lysis buffer containing 50 mM Tris-HCl (pH 7.5), 150 mM NaCl, 1% Triton X-100, protease inhibitor mixture (Complete EDTA-free, Roche) and phosphatase inhibitor cocktail mixture (PhosStop, Roche). The suspension was incubated at 4°C for 30 min and centrifuged at 20,000× *g* for 5 min. For immunoblot analysis, the supernatant was quantitated by the Bradford method (Coomassie PlusProtein Assay Reagent, Thermo Fisher Scientific) and denatured at 95°C for 5 min. The protein extracts were resolved by SDS-PAGE and transferred to a PVDF membrane (Immobilon-P, Millipore). Proteins were probed with primary antibodies against: mGluR1 N-terminus (kindly provided by Dr. Araishi, Kanazawa University Graduate School of Medicine, Kanazawa, Japan), HA (3F10, Roche), FLAG (Sigma, cat. no. A8592, RRID:AB_439702) or β-actin (Sigma, cat. no. A2228, RRID:AB_476697) at 4°C overnight. Antigen-antibody complexes were detected with secondary antibodies conjugated with HRP (Jackson Immunoresearch, cat. no. 123-065-021, RRID:AB_2314646) and visualized by enhanced chemiluminescence (Amersham Hyperfilm ECL, GE Healthcare). Co-immunoprecipitations were carried out based on previously described procedures (Ohtani et al., 2014). Briefly, isolated cerebella were homogenized in lysis buffer in which 1% Triton X-100 was replaced with 0.5% NP-40. The supernatant was incubated at 4°C overnight with a rat monoclonal antibody against the mGluR1 N-terminus (Hirata et al., 2012) or rat IgG coupled to Sepharose beads (GE Healthcare). After washing the Sepharose with lysis buffer, bound proteins were eluted with SDS-PAGE sample buffer. Proteins were subjected to immunoblot analysis using antibodies against Homer (Santa Cruz, cat. no. sc-8921, RRID:AB_648368), GluRδ2 (Chemicon, cat. no. AB1514) and TRPC3 (Alomone Labs, cat. no. ACC016, RRID:AB_2040236).

### Immunofluorescence

For immunofluorescence experiments, mice were deeply anesthetized with sodium pentobarbital (100 mg/kg body weight, i.p.) and then perfused transcardially with a fixative containing 4% paraformaldehyde in 0.1 M phosphate buffer (PB; pH 7.4). Brains were quickly extracted from the skull, cryoprotected with 30% sucrose in 0.1 M PB and sectioned at 40 μm thickness using a freezing microtome (FX-801, Yamato). Slices were blocked with a buffer containing 3% normal goat serum and 0.1% Triton X-100 in PBS and incubated with a primary antibody raised against the mGluR1 N-terminus (kindly provided by Dr. Araishi) at 4°C overnight. The antigen-primary antibody complex was visualized using an Alexa488-conjugated secondary antibody (Thermo Fisher Scientific). The sections were observed with a fluorescence microscope (BZ-8000, KEYENCE).

For double immunofluorescence experiments, brains were post-fixed in 4% paraformaldehyde for 3 h. Floating parasagittal sections of the cerebellum were cut at a thickness of 50 μm on a vibratome (VT1000, Leica Biosystems). Sections were permeabilized with 0.3% Triton X-100 in PBS for 30 min, followed by an incubation with 5% normal donkey serum in PBS. Sections were incubated with combinations of anti-calbindin (Frontier Institute, cat. no. Calbindin-GP-AF280, RRID:AB_2571570) and anti-mGluR1α (Frontier Institute, cat. no. mGluR1a-Rb-Af811, RRID:AB_2571799) antibodies. The immunoreaction was visualized using species-specific secondary antibodies conjugated with Alexa-488 or Alexa-594 (Invitrogen). Sections were counter-stained with 4’, 6-diamidino-2-phenylindole (DAPI, Roche) and examined with a confocal laser-scanning microscope (LSM 710, Zeiss).

### Freeze-Fracture Replica Immunogold Labeling

Mice were perfused transcardially with a fixative containing 1% paraformaldehyde and 15% saturated picric acid in 0.1 M PB at a rate of 5 mL/min for 10 min. The cerebellum was quickly extracted and sliced at 140 μm thickness on a vibratome (VT1000S, Leica). Slices were cryoprotected in 30% glycerol in 0.1 M PB and processed for freeze-fracture replica immunogold labeling (FRIL) as previously described (Mansouri et al., 2015). Briefly, sections were high-pressure frozen with a HPM 010 machine (Bal-Tec), freeze-fractured at −115°C and replicated with a first layer of 5 nm-thick carbon, shadowed by 2 nm-thick platinum followed by a second carbon layer of 15 nm in thickness in a freeze-etching BAF 060 device (Bal-Tec). The tissue attached to the replica was solubilized with shaking at 80°C for 20 h in a solubilization solution (pH 8.3) containing 15 mM Tris, 20% sucrose and 2.5% sodium dodecyl sulfate (SDS). Before the immunostaining, the solubilization solution was progressively replaced with 5% polyethylene glycol (PEG-6,000, Merck) in TBS. The replicas were blocked with a solution containing 5% bovine serum albumin (BSA) in TBS, and then incubated with primary antibodies at 15°C for 72 h. To detect mGluR1 on the exoplasmic face (E-face) a rabbit polyclonal antibody raised against the mGluR1 N-terminus (Ferraguti et al., 1998) was used at a dilution of 1:50, whereas on the protoplasmic face (P-face) a guinea pig polyclonal antibody raised against the mGluR1α C-terminus (Frontier Institute, cat. no. mGluR1a-Gp-Af660-1, RRID:AB_2571801) was used at a dilution of 1:500. Some replicas were double-labeled for GluRδ2: for the E-face, we used a guinea pig polyclonal antibody against the N-terminus diluted 1:100 (kindly provided by Prof. R. Shigemoto, Institute of Science and Technology Austria, Klosterneuburg, Austria); for the P-face, we used a rabbit polyclonal antibody raised against the C-terminus diluted 1:100 (Frontier Institute, cat. no. GluRd2C-Rb-Af500-1, RRID:AB_2571600). The replicas were then reacted with gold-conjugated species-specific secondary antibodies purchased from BBI solutions (10 and 15 nm) at 15°C overnight. The replicas were mounted on pioloform-coated mesh copper grids and observed in a transmission electron microscope (CM120, Philips) equipped with a Morada CCD transmission EM camera (Soft Imaging Systems).

### Sampling and Analysis of Gold Particles

To establish the distribution of mGluR1 in relation to the postsynaptic density (PSD), defined as a cluster of intramembrane particles (IMP) on the E-face, we measured the closest distance from the center of each gold particle to the PSD edge using the ImageJ software. Values inside the PSD were considered negative and those outside positive. The particles located directly on the synaptic edge were given a value equal to zero. Results were plotted using Prism 7 software for Mac (GraphPad Software, Inc., La Jolla, CA, USA). The frequency of immunogold particles was measured in 60 nm wide bins, keeping the edge of the synapse as 0.

### Data Analysis

Two-way ANOVA followed by the *post hoc* Tukey’s multiple comparison test was used to analyze rotarod and gold particle distance from the PSD edge experiments. The Chi-square test was used to test whether the frequency of synapses containing different amounts of gold particles for mGluR1 differed between mGluR1a-P/E-rescue and WT mice. Data were considered significant when *p* < 0.05.

## Results

### Generation of mGluR1γ-Tagged KI Mice and Its Expression in Cerebellum

**Figure 1:**
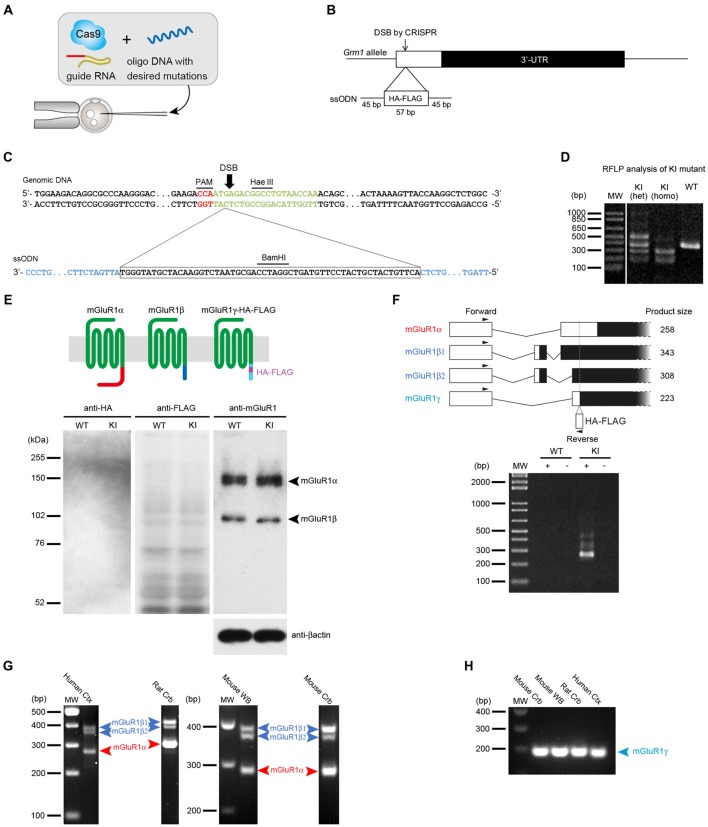
Generation of metabotropic glutamate receptors (mGluR1γ)-tagged knock-in (KI) mice and expression of mGluR1γ in mouse cerebellum. **(A)** Graphic representation of the CRISPR/Cas9 approach. To generate the mGluR1γ-tagged KI mice, single stranded oligodeoxynucleotide (ssODN) with the desired sequences was microinjected into wild type (WT) fertilized eggs together with Cas9 protein and crRNA/tracrRNA. **(B)** Targeting strategy to KI the HA and FLAG tags at the *Grm1* locus. The double-strand break (DSB) was introduced by CRISPR/Cas9 and the 57-base HA/FLAG sequence flanked by 45-base homology arms was inserted into exon X of *Grm1*. **(C)** Genomic DNA sequence around the KI site. The upper and lower sequences depict the genomic DNA and ssODN, respectively. The PAM sequence is shown in red, target sequence of crRNA in green and homology arms in blue. **(D)** Restriction fragment length polymorphism (RFLP) analysis of PCR products amplified from WT and mGluR1γ-tagged KI heterozygous (het) and homozygous (homo) mice. PCR fragments were digested with BamHI, and tag-insertion was confirmed by the presence of cleaved fragments by BamHI digestion. The expected size of BamHI-digested fragments is 389 and 9 bp in the WT allele, and 279, 167 and 9 bp in the KI allele. The largest fragment observed in the KI heterozygote lane may be derived from heteroduplex formation. **(E)** Expression of mGluR1 variants in WT and mGluR1γ-tagged KI mice. The diagram on the top schematically shows mGluR1 splice variants differing in their C-terminal domains (shown here in different colors). Protein extracts from the cerebella of WT and mGluR1γ-tagged homozygous KI mice were immunoblotted with antibodies against HA, FLAG, mGluR1 extracellular domain and β-actin. For anti-mGluR1, mGluR1α and mGluR1β are detected at around 150 and 100 kDa, respectively (right). No traces of mGluR1 variants were detected using HA (left) and FLAG (center) antibodies. **(F)** Reverse transcription PCR analysis of mGluR1 mRNA in cerebella of WT and mGluR1γ-tagged homozygous KI mice. The upper diagram shows alternative splicing patterns with locations of primers and expected sizes of PCR products. No detectable bands were observed at an expected size of the PCR product from mGluR1γ transcript. + and—indicate PCR analyses with and without reverse transcriptase, respectively. **(G)** Reverse transcription PCR analysis of mGluR1 splice variants mRNA in different species. Products corresponding only to mGluR1α and mGluR1β (β1 and β2; Ferraguti et al., 2008) could be amplified from human neocortex complementary DNA (cDNA), rat cerebellar cDNA, mouse whole brain and cerebellar cDNAs. Species-specific primers were used which gave rise to products of different lengths. **(H)** mGluR1γ transcripts could be amplified from all species by means of a nested PCR approach using as template gel-eluted bands between 200 bp and 400 bp from the first round of RT-PCRs.

To examine the expression of mGluR1γ in mouse cerebellum, the HA and FLAG tags were inserted in frame with the sequence coding for mGluR1γ in exon X of the mouse *Grm1* using the CRISPR/Cas9 system (Figures 1A,B). In transcripts generated from the splice site coding for mGluR1α, because of the different frame, the sequences corresponding to the tags were translated into amino acids unrelated to HA and FLAG. To generate mGluR1γ-tagged KI mice, a ssODN carrying the HA and FLAG tags, including a BamHI restriction site between the two tags (Figure 1C), was constructed and injected into fertilized eggs together with the Cas9 protein, crRNA and tracrRNA. Restriction fragment length polymorphism (RFLP) analysis demonstrated that the tags sequence was indeed introduced into the *Grm1* allele (Figure 1D). The precise integration of the tags was also confirmed by DNA sequencing (see Supplementary Figure S1). To assess the expression levels of mGluR1γ-HA/FLAG in these KI mice, cerebellar protein extracts were immunoblotted with antibodies against the N-terminal domain of mGluR1, as well as against the HA and FLAG tags (Figure 1E). Two bands at approximately 145 and 97 kDa, in good agreement with the molecular weight of mGluR1α and mGluR1β respectively, were detected in both WT and mGluR1γ-tagged KI mice (Figure 1E), whereas no bands could be observed compatible with mGluR1γ-HA/FLAG. Immunoblotting against HA and FLAG also did not reveal any specific band in mGluR1γ-tagged KI cerebellar extracts (Figure 1E). To test for the presence of mGluR1γ transcripts, we carried out PCR analyses on cerebellar cDNA libraries generated from WT and mGluR1γ-tagged KI mice. Using a reverse primer designed within the tags sequence, we were able to amplify several products from the cDNA of mGluR1γ-tagged KI mice only, as expected. The observed bands correspond in length to mGluR1α and mGluR1β variants, whereas PCR products from mGluR1γ transcripts were not detected in mGluR1γ-tagged KI mice (Figure 1F). The absence of a detectable band corresponding to mGluR1γ suggested two possible scenarios: *(a)* that the expression of such variant is extremely low, or *(b)* that this splice variant is not transcribed at all in mice, as in this species it was never reported so far. To resolve this issue, we performed nested PCRs on cDNA libraries from rat cerebellum and human cerebral cortex, in which mGluR1γ could be previously amplified (Laurie et al., 1996; Mary et al., 1997; Soloviev et al., 1999) as well as from mouse cerebellum and whole brain. While no mGluR1γ band could be detected in the first PCR amplification (Figure 1G), the use of nested primers was able to reveal mGluR1γ in all libraries and species (Figure 1H). Taken together, these data indicate that mGluR1γ is transcribed only at very low levels, but it is most likely not translated or at levels that remain undetectable by immunoblotting.

### Generation of mGluR1a-P/E-Rescue Mice Carrying a Point Mutation in the Homer Binding Domain

**Figure 2:**
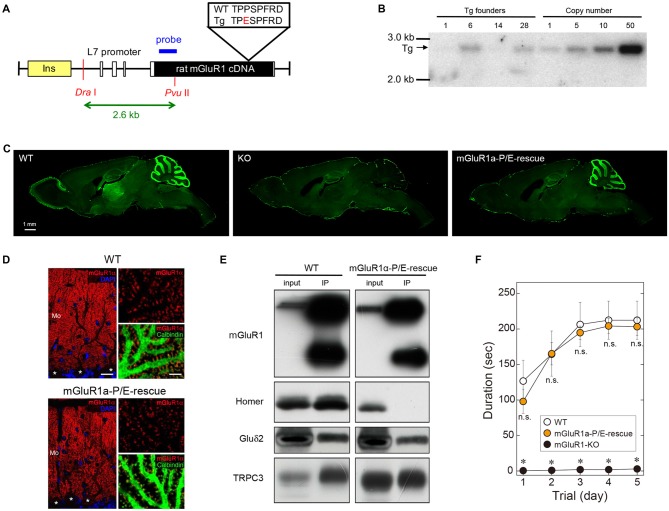
Generation of mGluR1a-P/E-rescue mice. **(A)** Schematic drawing of the L7-mGluR1a-P/E transgene construct. Proline 1153 was replaced by a glutamate residue in the Homer binding domain of mGluR1α. Rat mGluR1α-P/E cDNA was inserted into the L7 promoter vector. Open and yellow boxes represent exons of the L7 gene and an insulator sequence, respectively. The expected size of *Dra* I-*Pvu* II restriction fragment from the transgene is shown below the drawing. **(B)** Southern blot analysis of genomic DNA isolated from tail biopsies of hemizygous L7-mGluR1a-P/E transgenic (Tg) mice. Genomic integration of the transgene was confirmed by the presence of a 2.6-kb *Dra* I-*Pvu* II fragment. Among four founder mice, #28 line was intercrossed to generate homozygous Tg mice for further experiments. **(C)** Immunofluorescence analysis of mGluR1 protein expression. Parasagittal sections from WT (left), mGluR1-knock out (KO; middle) and mGluR1a-P/E-rescue (right) mice were stained with an antibody against the extracellular domain of mGluR1. Scale bar, 1 mm. **(D)** Double immunofluorescence analysis of mGluR1α expression in cerebellar Purkinje cells (PCs; somata indicated by *) of 10 weeks-old WT (upper) and mGluR1a-P/E-rescue (lower) mice. Sections were stained with antibodies against mGluR1α (RED), calbindin (green) and counterstained with DAPI (blue). Mo, molecular layer. Scale bars, 20 μm (left panels) and 5 μm (right panels). **(E)** Immunoprecipitation analysis of the mGluR1 protein complex. GluRδ2 and transient receptor potential channel 3 (TRPC3), but not long Homers, were immunoprecipitated (IP) by the mGluR1 antibody from mouse cerebellar protein extracts, confirming that the P/E mutation indeed prevents *in vivo* the interaction between the C-terminal tail of mGluR1α and long Homers. **(F)** Rotarod task in WT (*n* = 4), mGluR1a-P/E-rescue (*n* = 6) and mGluR1-KO (*n* = 6) mice. Each mouse was subjected to three training sessions per day for 5 days on the accelerating rotarod (4–40 rpm over 300 s). No significant differences were detected between WT and mGluR1a-P/E-rescue mice (Two-way ANOVA, interaction *F*_(8,65)_ = 1.389, *p* = 0.2181), whereas mGluR1-KO mice were unable to remain on the rod (genotype *F*_(2,65)_ = 122.8, *p* < 0.0001) consistent with previous studies (Aiba et al., 1994). *p < 0.01 (Two-way ANOVA followed by Tukey’s multiple comparisons). n.s., not significant.

Trafficking and subcellular localization of mGluR1 have been postulated to be dependent, at least in part, on long Homer proteins (Xiao et al., 1998; Tu et al., 1999; Ciruela et al., 2000). We have previously shown that disrupting the interaction between mGluR1 and long Homers by means of the dominant-negative TAT-Homer1a did not significantly alter the subsynaptic distribution of mGluR1 (Mansouri et al., 2015). However, the approach had several caveats as TAT-Homer1a might have not been sufficiently effective to disrupt the mGluR1-Homer complex, or the fraction of dissociated mGluR1α could have not been large enough to be detected. Therefore, to further address the issue of the role of Homer binding to mGluR1 in the subcellular localization of this receptor, we generated a mouse line carrying a point mutation which prevents mGluR1 from binding to Homer proteins (Figure 2). Proline 1153 in the Homer binding domain of the mGluR1α intracellular C-terminus was replaced by a glutamate residue. We introduced the L7-mGluR1a-P/E transgene, that expresses the mutant mGluR1α under the control of the PC-specific L7 promoter, into mGluR1-KO mice (Figure 2A). Among four L7-mGluR1a-P/E Tg founder mice, line #28 was used for subsequent analyses (Figure 2B). Hereafter, we refer to mGluR1-KO mice carrying this transgene as mGluR1a-P/E-rescue mice. Immunofluorescence analysis confirmed the PC-specific expression of the mGluR1α-P/E protein, which was highly comparable to WT animals (Figures 2C,D). Moreover, while GluRδ2 and TRPC3 were effectively co-immunoprecipitated (IP) with mGluR1 from cerebellar membranes of mGluR1a-P/E-rescue mice, using an anti-N-terminus mGluR1 antibody, Homer proteins were not detected in the mGluR1 complex (Figure 2E), confirming the critical role of proline 1153 for Homer binding *in vivo*. mGluR1a-P/E-rescue mice did not show any evident behavioral phenotype when tested in the accelerating rotarod test (Figure 2F). These results suggest that motor incoordination of mGluR1-KO mice can be rescued by the PC-specific expression of mGluR1α-P/E.

### Subsynaptic mGluR1 Localization at Parallel Fiber-Purkinje Cell Synapses

**Figure 3:**
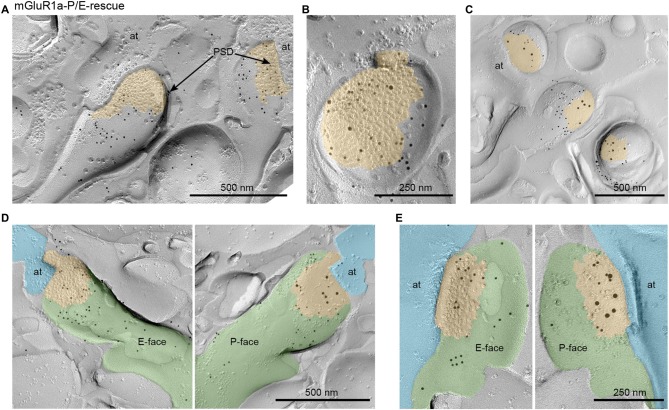
Subcellular localization of mGluR1 at parallel fiber (PF)-PC synapses in mGluR1a-P/E-rescue mice detected by means of the fracture replica immunogold labeling (FRIL) technique. **(A)** Representative micrograph of PC spines forming synaptic contacts with axon terminals (at) of PF immunolabeled with an antibody against the mGluR1 extracellular domain and visualized with secondary antibodies conjugated with 10 nm gold particles. The spine on the left shows immunoparticles located primarily outside the postsynaptic density (PSD) and extra-synaptically, whereas the nearby spine on the right side shows substantial intrasynaptic labeling. Scale bar, 500 nm. **(B)** The exoplasmic face (E-face) of a PC spine is double labeled with antibodies against the extracellular N-terminal domain of mGluR1 (5 nm) and GluRδ2 (10 nm). In this micrograph, the intrasynaptic location of mGluR1 can be clearly observed. Scale bar, 250 nm. **(C)** The protoplasmic face (P-face) of PC spines is double labeled with antibodies against the intracellular C-terminal domain of mGluR1α (5 nm) and GluRδ2 (10 nm). The P-face mGluR1 labeling pattern was consistent with the one observed on the E-face demonstrating a highly variable intrasynaptic localization of mGluR1. Scale bar, 500 nm. **(D,E)** Two examples showing the two faces of the same PF-PC synapse labeled for mGluR1 (5 nm) on the E-face and mGluR1α (5 nm) and GluRδ2 (10 nm) on the P-face confirming that, in a subset of synapses, the loss of the interaction between mGluR1α and long Homers allows lateral mobility into the PSD of mGluR1. Scale bars, **(D)** 500 nm; **(E)** 250 nm. Pseudocolors have been used to simplify the identification of the same structure: PC spines are shown in green, PF axon terminals in light blue and PSD in orange.

We further examined the subsynaptic localization of mGluR1 at PF-PC synapses in mGluR1a-P/E-rescue mice using the FRIL technique. PF-PC synapses were identified on E-face of replicas as GluRδ2-labeled IMP clusters. Despite qualitative analysis of the subcellular distribution of mGluR1α-P/E showed a prevalent perisynaptic localization similar to that previously observed in WT mice (Mansouri et al., 2015), we observed in these animals substantial intrasynaptic labeling in a subset of synapses (Figures 3A–C). Indeed, when we compared the frequency of synapses containing intrasynaptic gold particles between WT and mGluR1a-P/E-rescue mice, we observed that in the latter many more synapses had ≥3 particles (Chi square *p* < 0.0001; Table 1). This finding was further confirmed by analyzing the P-face of the very same synapses labeled with antibodies against GluRδ2 and mGluR1α intracellular epitopes (Figures 3D,E).

**Table 1:** Frequency of synapses categorized based on the number of gold immunoparticles inside the postsynaptic density (PSD) of parallel fiber (PF)-Purkinje cell (PC) synapses.

Intrasynaptic particles	0	1	2	≥3
WT	57 (44%)	31 (24%)	15 (11%)	27 (21%)
mGluR1a-P/E-rescue	24 (22%)	20 (18%)	22 (20%)	44 (40%)

**Figure 4:**
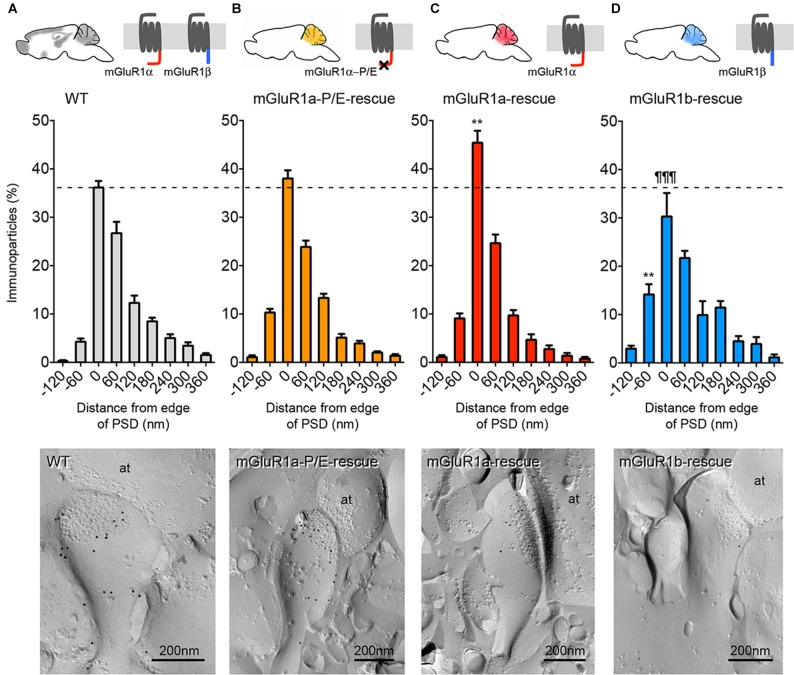
Comparison of the frequency distribution of mGluR1 at different subsynaptic areas of PF-PC synapse in different mGluR1-rescue mouse lines. Frequency distribution of mGluR1 at PF-PC synapses in WT (**A**; *n* = 130 synapses obtained from five mice, 1359 particles analyzed), mGluR1a-P/E-rescue (**B**; *n* = 110 synapses obtained from five mice, 1307 particles analyzed), mGluR1a-rescue (**C**; *n* = 118 synapses obtained from five mice, 920 particles analyzed) and mGluR1b-rescue (**D**; *n* = 131 synapses obtained from five mice, 586 particles analyzed) mice. Histograms represent 60 nm-wide bins, as used in a previous report (Mansouri et al., 2015). **p < 0.01 vs. WT; ^¶¶¶^* p* < 0.0001 vs. mGluR1a-rescue (Two-way ANOVA followed by Tukey’s multiple comparisons). The bottom panels are representative electron micrographs (E-face) from each mouse line labeled for mGluR1 (10 nm gold particles). It can be appreciated that the mGluR1 labeling density at the plasma membrane in mGluR1a- and mGluR1b-rescue mice is significantly lower in comparison to WT animals. Scale bars, 200 nm. Abbreviation: at, PF axon terminal.

In order to more quantitatively compare the distribution of mGluR1 relative to the postsynaptic specialization at PF-PC synapses between mGluR1a-P/E-rescue and WT mice, we measured the distance between gold particles and the edge of the synapse. We extended our quantitative analysis also to mGluR1a-rescue (Ichise et al., 2000) and mGluR1b-rescue mice (Ohtani et al., 2014) as in these mouse lines a diverse subsynaptic distribution of mGluR1 was previously reported (Ohtani et al., 2014). Two-way ANOVA showed a positive genotype × distance of gold particle from PSD interaction (*F*_(24,144)_ = 4.441, *p* < 0.0001). Tukey’s multiple comparison analysis revealed that the mGluR1 frequency distribution in mGluR1a-P/E-rescue mice (*n* = 110 synapses) was similar to WT animals (*n* = 130 synapses; Figures 4A,B), whereas a significant difference was observed between WT and mGluR1a-rescue (*n* = 118 synapses) mice in the fraction of gold particles at the synaptic rim, namely in the interval between −30 nm to +30 nm (*p* < 0.0078; Figures 4A,C), and between WT and mGluR1b-rescue (*n* = 131 synapses) mice in the interval between −90 nm to −30 nm (*p* < 0.0024; Figures 4A,D). A highly significant difference (*p* < 0.0001) was also detected between mGluR1a-rescue and mGluR1b-rescue mice in the −30 to +30 nm interval (Figures 4C,D).

**Figure 5:**
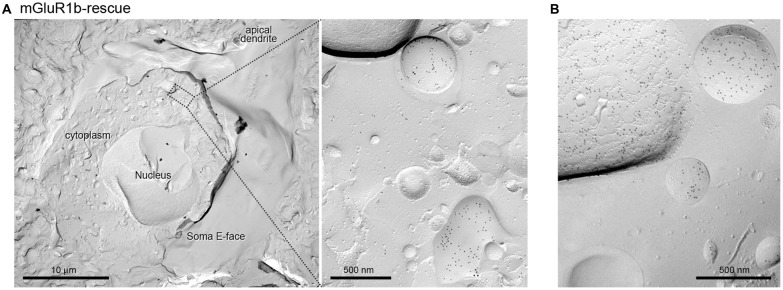
Intracellular organelles of PCs in mGluR1b-rescue mice are enriched in mGluR1. **(A)** Cross-fracture of a PC soma revealing the nucleus and several intracellular organelles. The membrane of several vesicles of the smooth endoplasmatic reticulum, as shown in the inset **(A)**, and of other unidentified intracellular organelles **(B)** were heavily immunolabeled for mGluR1. Scale bars, **(A)** 10 μm, Inset 500 nm, **(B)** 500 nm.

While analyzing the subcellular distribution of mGluR1β, we observed that the vast majority of gold particles did not reach the plasma membrane, but accumulated at the membrane of intracellular organelles, most likely vesicles of the smooth endoplasmatic reticulum, within PC dendrites and somata (Figure 5).

## Discussion

Our study sets several firm points on matters which have been debated in the field over many years. First, only two transmembrane mGluR1 isoforms are present at cerebellar synapses, namely mGluR1α and mGluR1β. The short variant mGluR1γ is transcribed at very low levels and is either untranslated or in amounts that are undetectable with our current techniques in the mouse cerebellum. Second, disruption of the interaction between mGluR1α and long Homer proteins through a point mutation, despite it does not dramatically change the perisynaptic distribution of this receptor, it facilitates under circumstances that are at present unknown its diffusion within the PSD at PF-PC synapses. Third, in mGluR1a-rescue mice, which express only the mGluR1α isoform in PC, mGluR1 accumulates closer to the edge of the PSD as compared to WT control mice. Conversely, in mGluR1b-rescue mice, the subsynaptic localization of the mGluR1β isoform shows a less pronounced perijunctional distribution as well as high accumulation in intracellular organelles, demonstrating an impaired trafficking to the plasma membrane.

Since its cloning in 1996 (Laurie et al., 1996), the functional significance of the short mGluR1γ variant has remained elusive. To this, it has largely contributed the uncertainty about the pattern and levels of expression of this receptor isoform. Many laboratories, including ours, have tried to develop antibodies against the specific C-terminal domain of mGluR1γ without success. Multiple explanations were put forward for these failures, such as a limited antigenicity of the epitope sequence. Abundant mGluR1γ mRNA expression in PC was reported by *in situ* hybridization (Berthele et al., 1998), whereas PCR analysis showed a significantly lower abundance of mGluR1γ transcripts compared to mGluR1α and mGluR1β in the cerebellar cortex (Mary et al., 1997; Soloviev et al., 1999). Our findings are fully consistent with these latter studies proving a very low transcriptional expression of mGluR1γ. Moreover, we could demonstrate a lack of detectable levels of translated mGluR1γ since none of the two epitope tags, inserted in frame at the C-terminus of this variant, could be revealed by western immunoblotting. Therefore, we conclude that only the mGluR1α and mGluR1β variants are present at cerebellar synapses.

Previous studies have suggested that long Homer proteins play a role in the trafficking and accumulation of group I mGluRs near to synaptic sites (Tadokoro et al., 1999; Tu et al., 1999; Sergé et al., 2002). However, given the largely non-overlapping subcellular distribution of the two proteins (Baude et al., 1993; Nusser et al., 1994; Tao-Cheng et al., 2014; Mansouri et al., 2015), interactions between long Homers and mGluR1α *in vivo* should be limited to the very edge of PSDs. Indeed, we have observed that in mGluR1a-rescue mice a higher proportion of gold immunoparticles identifying mGluR1 was present in the 60 nm spanning the PSD edge compared to WT mice. The broader distribution of mGluR1 in WT mice suggests that the presence of mGluR1β and the potential formation of heterodimeric complexes with mGluR1α may anchor these receptors to other scaffolding proteins present in perisynaptic sites besides Homer proteins. We directly tested the potential requirement of long Homers to anchor mGluR1α at perisynaptic sites by developing a novel mouse line in which mGluR1α carries a point mutation preventing its binding to Homer proteins. In PF-PC synapses, the P1153E mutation did not significantly alter the frequency distribution of mGluR1α-P/E at perisynaptic sites. In line with this, mGluR1a-P/E-rescue mice showed a normal motor coordination in the accelerating rotarod test. These findings are consistent with our previous work in which the membrane-permeable dominant-negative TAT-Homer1a, used to disrupt the binding between mGluR1 and long Homer proteins, did not change the subsynaptic distribution of mGluR1 (Mansouri et al., 2015). However, in a limited fraction of PF-PC synapses in mGluR1a-P/E-rescue mice we could detect a relatively high density of intrasynaptic mGluR1, suggesting that the lateral mobility of mGluR1α is increased by disrupting its interaction with long Homers. Why this was not observed in the majority of synapses is unclear, but we could surmise that the membrane trafficking of mGluR1α involves a number of interacting proteins regulated by synaptic activity, e.g., Preso1 (Hu et al., 2012) and Norbin (Wang et al., 2009), limiting the impact of the long Homers interaction. In general, the targeting of GPCR to the plasma membrane as well as in or around the PSD is a highly complex and regulated process that most likely involves dozens of interacting and scaffolding proteins for each GPCR (Bernard et al., 2006; Dunn and Ferguson, 2015). It is, therefore, not entirely surprising that disrupting a single interaction between an integral membrane receptor and a scaffolding protein does not affect, or only modestly, the receptor synaptic localization. In summary, our results corroborate the theory that the interaction between mGluR1α and long Homer proteins facilitates the synaptic clustering of the receptor (Tadokoro et al., 1999; Tu et al., 1999), yet probably having only a limited role in the perisynaptic accumulation of these receptors *in vivo*, a mechanism most likely shared with many other interacting proteins.

Here, we show a weaker surface expression of mGluR1β in mGluR1b-rescue mice, confirming earlier studies (Chan et al., 2001; Kumpost et al., 2008; Ohtani et al., 2014), as well as a less pronounced but still evident perisynaptic clustering of this receptor variant, also consistent with previous data (Mateos et al., 2000; Ohtani et al., 2014; Techlovská et al., 2014). The prominent intracellular retention of mGluR1β, when expressed in the absence of other group I mGluRs with long C-terminal domains, is in line with the proposed retention role of the RRKK domain present in its C-terminal tail that prevents the trafficking of the receptor from cis-Golgi to the ER (Ciruela et al., 2000; Chan et al., 2001; Kumpost et al., 2008). Heterodimerization between mGluR1α and mGluR1β neutralizes the RRKK motif promoting the trafficking of the heterodimer to the cell surface (Kumpost et al., 2008). The presence of heterodimers in WT animals may explain the in-between distribution observed at PF-PC synapses in these animals in comparison to mGluR1a- and mGluR1b-rescue mice. On the other hand, in mGluR1b-rescue mice sufficient surface expression of mGluR1β was achieved to normalize slow excitatory postsynaptic potentials at PF-PC synapses and to rescue the motor coordination deficits of mGluR1-KO mice (Ohtani et al., 2014). This suggests that compensatory or supplementary mechanisms for the trafficking to the plasma membrane and to synapses of mGluR1β exist, but with a considerably lower efficacy.

In conclusion, this study demonstrates that mGluR1γ is not translated at detectable levels in PCs, hence the participation of mGluR1 splice variants in cerebellar physiology remains a “menage a deux” between mGluR1α and mGluR1β. The perisynaptic distribution of mGluR1 is highly dependent on the different C-terminal domains of mGluR1α and β, and although long Homer proteins can in part influence the lateral mobility of mGluR1 containing the α isoform, it remains to be determined which and how additional adaptor proteins participate to the membrane trafficking of this receptor.

## Author Contributions

RN, CS, HK, AA and FF conceived and designed the project. HK and KN generated the mGluR1γ-KI mice. HK and KN generated the mGluR1-P/E-rescue mouse line. RN, MF, SS and YS were involved with experimental and analytical aspects of the manuscript. RN and FF performed statistical analyses. RN, AA and FF wrote the manuscript. All contributing authors commented on the manuscript.

## Conflict of Interest Statement

The authors declare that the research was conducted in the absence of any commercial or financial relationships that could be construed as a potential conflict of interest.

## Supplementary Material

FIGURE S1 Electropherogram of the Sanger DNA sequencing of exon X of mGluR1γ-tagged KI mice encompassing the splice site used to transcribe mGluR1α, the HA and FLAG tags and the BamHI restriction site.

## References

[B1] Aiba A. Kano M. Chen C. Stanton M. E. Fox G. D. Herrup K.. (1994). Deficient cerebellar long-term depression and impaired motor learning in mGluR1 mutant mice. Cell 79, 377–388. 10.1016/0092-8674(94)90205-4 7954803

[B2] Aramori I. Nakanishi S. (1992). Signal transduction and pharmacological characteristics of a metabotropic glutamate receptor, mGluR1, in transfected CHO cells. Neuron 8, 757–765. 10.1016/0896-6273(92)90096-v 1314623

[B3] Baude A. Nusser Z. Roberts J. D. Mulvihill E. McIlhinney R. A. Somogyi P. (1993). The metabotropic glutamate receptor (mGluR1 α) is concentrated at perisynaptic membrane of neuronal subpopulations as detected by immunogold reaction. Neuron 11, 771–787. 10.1016/0896-6273(93)90086-7 8104433

[B4] Bernard V. Décossas M. Liste I. Bloch B. (2006). Intraneuronal trafficking of G-protein-coupled receptors *in vivo*. Trends Neurosci. 29, 140–147. 10.1016/j.tins.2006.01.006 16443287

[B5] Berthele A. Laurie D. J. Platzer S. Zieglgänsberger W. Tölle T. R. Sommer B. (1998). Differential expression of rat and human type I metabotropic glutamate receptor splice variant messenger RNAs. Neuroscience 85, 733–749. 10.1016/s0306-4522(97)00670-2 9639268

[B6] Brakeman P. R. Lanahan A. A. O’Brien R. Roche K. Barnes C. A. Huganir R. L.. (1997). Homer: a protein that selectively binds metabotropic glutamate receptors. Nature 386, 284–288. 10.1038/386284a0 9069287

[B7] Chan W. Y. Soloviev M. M. Ciruela F. McIlhinney R. A. (2001). Molecular determinants of metabotropic glutamate receptor 1B trafficking. Mol. Cell. Neurosci. 17, 577–588. 10.1006/mcne.2001.0965 11273651

[B8] Ciruela F. Soloviev M. M. Chan W. Y. McIlhinney R. A. (2000). Homer-1c/Vesl-1L modulates the cell surface targeting of metabotropic glutamate receptor type 1α: evidence for an anchoring function. Mol. Cell. Neurosci. 15, 36–50. 10.1006/mcne.1999.0808 10662504

[B9] Conquet F. Bashir Z. I. Davies C. H. Daniel H. Ferraguti F. Bordi F.. (1994). Motor deficit and impairment of synaptic plasticity in mice lacking mGluR1. Nature 372, 237–243. 10.1038/372237a0 7969468

[B10] Corti C. Restituito S. Rimland J. Brabet J. Corsi M. Pin J.-P.. (1998). Cloning and characterization of alternative mRNA forms for the rat metabotropic glutamate receptors mGluR7 and mGluR8. Eur. J. Neurosci. 10, 3629–3641. 10.1046/j.1460-9568.1998.00371.x 9875342

[B11] Crepaldi L. Lackner C. Corti C. Ferraguti F. (2007). Transcriptional activators and repressors for the neural-specific expression of a metabotropic glutamate receptor. J. Biol. Chem. 282, 17877–17889. 10.1074/jbc.M700149200 17430891

[B12] Dunn H. A. Ferguson S. S. (2015). PDZ protein regulation of G protein-coupled receptor trafficking and signaling pathways. Mol. Pharmacol. 88, 624–639. 10.1124/mol.115.098509 25808930

[B13] Enz R. (2012). Structure of metabotropic glutamate receptor C-terminal domains in contact with interacting proteins. Front. Mol. Neurosci. 5:52. 10.3389/fnmol.2012.00052 22536173 PMC3332230

[B14] Ferraguti F. Conquet F. Corti C. Grandes P. Kuhn R. Knopfel T. (1998). Immunohistochemical localization of the mGluR1 β metabotropic glutamate receptor in the adult rodent forebrain: evidence for a differential distribution of mGluR1 splice variants. J. Comp. Neurol. 400, 391–407. 10.1002/(sici)1096-9861(19981026)400:3<391::aid-cne8>3.0.co;2-3 9779943

[B15] Ferraguti F. Crepaldi L. Nicoletti F. (2008). Metabotropic glutamate 1 receptor: current concepts and perspectives. Pharmacol. Rev. 60, 536–581. 10.1124/pr.108.000166 19112153

[B16] Finch E. A. Augustine G. J. (1998). Local calcium signalling by inositol-1,4,5-trisphosphate in Purkinje cell dendrites. Nature 396, 753–756. 10.1038/25541 9874372

[B17] Hartmann J. Blum R. Kovalchuk Y. Adelsberger H. Kuner R. Durand G. M.. (2004). Distinct roles of Gα_q_ and Gα_11_ for Purkinje cell signaling and motor behavior. J. Neurosci. 24, 5119–5130. 10.1523/JNEUROSCI.4193-03.2004 15175381 PMC6729195

[B18] Hartmann J. Dragicevic E. Adelsberger H. Henning H. A. Sumser M. Abramowitz J.. (2008). TRPC3 channels are required for synaptic transmission and motor coordination. Neuron 59, 392–398. 10.1016/j.neuron.2008.06.009 18701065 PMC2643468

[B19] Hirata T. Kumada T. Kawasaki T. Furukawa T. Aiba A. Conquet F.. (2012). Guidepost neurons for the lateral olfactory tract: expression of metabotropic glutamate receptor 1 and innervation by glutamatergic olfactory bulb axons. Dev. Neurobiol. 72, 1559–1576. 10.1002/dneu.22030 22539416

[B20] Hu J.-H. Yang L. Kammermeier P. J. Moore C. G. Brakeman P. R. Tu J.. (2012). Preso1 dynamically regulates group I metabotropic glutamate receptors. Nat. Neurosci. 15, 836–844. 10.1038/nn.3103 22561452 PMC3434267

[B21] Ichise T. Kano M. Hashimoto K. Yanagihara D. Nakao K. Shigemoto R.. (2000). mGluR1 in cerebellar Purkinje cells essential for long-term depression, synapse elimination and motor coordination. Science 288, 1832–1835. 10.1126/science.288.5472.1832 10846166

[B22] Kano M. Hashimoto K. Tabata T. (2008). Type-1 metabotropic glutamate receptor in cerebellar Purkinje cells: a key molecule responsible for long-term depression, endocannabinoid signalling and synapse elimination. Philos. Trans. R. Soc. Lond. B Biol. Sci. 363, 2173–2186. 10.1098/rstb.2008.2270 18339599 PMC2610189

[B23] Kumpost J. Syrova Z. Kulihova L. Frankova D. Bologna J. C. Hlavackova V.. (2008). Surface expression of metabotropic glutamate receptor variants mGluR1a and mGluR1b in transfected HEK293 cells. Neuropharmacology 55, 409–418. 10.1016/j.neuropharm.2008.06.073 18627772

[B24] Laurie D. J. Boddeke H. W. Hiltscher R. Sommer B. (1996). HmGlu1d, a novel splice variant of the human type I metabotropic glutamate receptor. Eur. J. Pharmacol. 296, R1–R3. 10.1016/0014-2999(95)00868-3 8838462

[B25] Mansouri M. Kasugai Y. Bertaso F. Fukazawa Y. Raynaud F. Perroy J.. (2015). Different subsynaptic localization of mGlu1 receptors at glutamatergic and GABAergic synapses in the rodent cerebellar cortex. Eur. J. Neurosci. 41, 157–167. 10.1111/ejn.12779 25377770

[B26] Mary S. Stephan D. Gomeza J. Bockaert J. Pruss R. M. Pin J. P. (1997). The rat mGlu1d receptor splice variant shares functional properties with the other short isoforms of mGlu1 receptor. Eur. J. Pharmacol. 335, 65–72. 10.1016/s0014-2999(97)01155-2 9371547

[B27] Masu M. Tanabe Y. Tsuchida K. Shigemoto R. Nakanishi S. (1991). Sequence and expression of a metabotropic glutamate receptor. Nature 349, 760–765. 10.1038/349760a0 1847995

[B28] Mateos J. M. Benítez R. Elezgarai I. Azkue J. J. Lázaro E. Osorio A.. (2000). Immunolocalization of the mGluR1b splice variant of the metabotropic glutamate receptor 1 at parallel fiber-Purkinje cell synapses in the rat cerebellar cortex. J. Neurochem. 74, 1301–1309. 10.1046/j.1471-4159.2000.741301.x 10693964

[B29] Miyata M. Kim H. T. Hashimoto K. Lee T. K. Cho S. Y. Jiang H.. (2001). Deficient long-term synaptic depression in the rostral cerebellum correlated with impaired motor learning in phospholipase C β4 mutant mice. Eur. J. Neurosci. 13, 1945–1954. 10.1046/j.0953-816x.2001.01570.x 11403688

[B30] Nusser Z. Mulvihill E. Streit P. Somogyi P. (1994). Subsynaptic segregation of metabotropic and ionotropic glutamate receptors as revealed by immunogold localization. Neuroscience 61, 421–427. 10.1016/0306-4522(94)90421-9 7969918

[B31] Oberdick J. Smeyne R. J. Mann J. R. Zackson S. Morgan J. I. (1990). A promoter that drives transgene expression in cerebellar Purkinje and retinal bipolar neurons. Science 248, 223–226. 10.1126/science.2109351 2109351

[B32] Ohtani Y. Miyata M. Hashimoto K. Tabata T. Kishimoto Y. Fukaya M.. (2014). The synaptic targeting of mGluR1 by its carboxyl-terminal domain is crucial for cerebellar function. J. Neurosci. 34, 2702–2712. 10.1523/JNEUROSCI.3542-13.2014 24523559 PMC6802745

[B33] Pin J.-P. Bettler B. (2016). Organization and functions of mGlu and GABA_B_ receptor complexes. Nature 540, 60–68. 10.1038/nature20566 27905440

[B34] Sergé A. Fourgeaud L. Hémar A. Choquet D. (2002). Receptor activation and homer differentially control the lateral mobility of metabotropic glutamate receptor 5 in the neuronal membrane. J. Neurosci. 22, 3910–3920. 10.1523/jneurosci.22-10-03910.2002 12019310 PMC6757631

[B35] Soloviev M. M. Ciruela F. Chan W. Y. McIlhinney R. A. (1999). Identification, cloning and analysis of expression of a new alternatively spliced form of the metabotropic glutamate receptor mGluR1 mRNA1. Biochim. Biophys. Acta 1446, 161–166. 10.1016/s0167-4781(99)00083-4 10395931

[B36] Suh Y. H. Chang K. Roche K. W. (2018). Metabotropic glutamate receptor trafficking. Mol. Cell. Neurosci. 91, 10–24. 10.1016/j.mcn.2018.03.014 29604330 PMC6128748

[B37] Tadokoro S. Tachibana T. Imanaka T. Nishida W. Sobue K. (1999). Involvement of unique leucine-zipper motif of PSD-Zip45 (Homer 1c/vesl-1L) in group 1 metabotropic glutamate receptor clustering. Proc. Natl. Acad. Sci. U S A 96, 13801–13806. 10.1073/pnas.96.24.13801 10570153 PMC24145

[B38] Takechi H. Eilers J. Konnerth A. (1998). A new class of synaptic response involving calcium release in dendritic spines. Nature 396, 757–760. 10.1038/25547 9874373

[B39] Tanabe Y. Masu M. Ishii T. Shigemoto R. Nakanishi S. (1992). A family of metabotropic glutamate receptors. Neuron 8, 169–179. 10.1016/0896-6273(92)90118-W 1309649

[B40] Tao-Cheng J.-H. Thein S. Yang Y. Reese T. S. Gallant P. E. (2014). Homer is concentrated at the postsynaptic density and does not redistribute after acute synaptic stimulation. Neuroscience 266, 80–90. 10.1016/j.neuroscience.2014.01.066 24530450 PMC3998121

[B41] Techlovská Š. Chambers J. N. Dvořáková M. Petralia R. S. Wang Y. X. Hájková A.. (2014). Metabotropic glutamate receptor 1 splice variants mGluR1a and mGluR1b combine in mGluR1a/b dimers *in vivo*. Neuropharmacology 86, 329–336. 10.1016/j.neuropharm.2014.08.011 25158311 PMC4188797

[B42] Tu J. C. Xiao B. Naisbitt S. Yuan J. P. Petralia R. S. Brakeman P.. (1999). Coupling of mGluR/Homer and PSD-95 complexes by the Shank family of postsynaptic density proteins. Neuron 23, 583–592. 10.1016/s0896-6273(00)80810-7 10433269

[B43] Tu J. C. Xiao B. Yuan J. P. Lanahan A. A. Leoffert K. Li M.. (1998). Homer binds a novel proline-rich motif and links group 1 metabotropic glutamate receptors with IP3 receptors. Neuron 21, 717–726. 10.1016/s0896-6273(00)80589-9 9808459

[B44] Wang H. Westin L. Nong Y. Birnbaum S. Bendor J. Brismar H.. (2009). Norbin is an endogenous regulator of metabotropic glutamate receptor 5 signaling. Science 326, 1554–1557. 10.1126/science.1178496 20007903 PMC2796550

[B45] Xiao B. Tu J. C. Petralia R. S. Yuan J. P. Doan A. Breder C. D.. (1998). Homer regulates the association of group 1 metabotropic glutamate receptors with multivalent complexes of homer-related, synaptic proteins. Neuron 21, 707–716. 10.1016/s0896-6273(00)80588-7 9808458

[B46] Zhu H. Ryan K. Chen S. (1999). Cloning of novel splice variants of mouse mGluR1. Mol. Brain Res. 73, 93–103. 10.1016/s0169-328x(99)00239-9 10581402

