# Supplementary figures and images for: New Features on the Expression and Trafficking of mGluR1 Splice Variants Exposed by Two Novel Mutant Mouse Lines
Source: Front Mol Neurosci. 2018 Dec 3;11:439. doi: 10.3389/fnmol.2018.00439 (PMC6287019; doi:10.3389/fnmol.2018.00439)

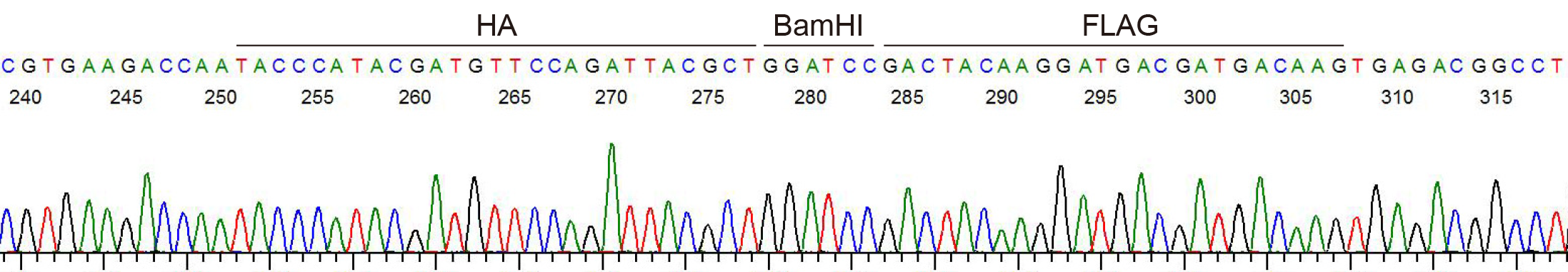

Supplement: FIGURE S1 — Electropherogram of the Sanger DNA sequencing of exon X of mGluR1γ-tagged KI mice encompassing the splice site used to transcribe mGluR1α, the HA and FLAG tags and the BamHI restriction site. [file Image_1.TIF]
